# Development of hand surgery services in Malaysia

**DOI:** 10.1186/1753-6561-9-S3-A1

**Published:** 2015-05-19

**Authors:** Abdul Hamid Abdul Kadir

**Affiliations:** 1 Assunta Hospital, Petaling Jaya, 46990, Malaysia

## 

Hand surgery in Malaysia began modestly with the correction of claw deformity in leprosy patients early in the last century, and began to evolve over the years into the treatment of injuries in plantation and industrial workers, at home and then in road crashes.

With training of some of our surgeons in various overseas centres, particularly in the UK and then in USA and Singapore, hand and microsurgery began to be developed in General Hospital Kuala Lumpur and University Malaya Hospital, when they returned home.

An international three-day conference on hand surgery in Kuala Lumpur in the mid-1980's created greater awareness on the topic and helped to bring hand surgery to the fore-front, with setting up hand clinics, training in micro-surgery, and so on, in the major hospitals in Kuala Lumpur initially.

Today, there are dedicated hand surgeons, technicians and hand therapists in the major government and private hospitals throughout the country, providing excellent services in this specialised and demanding area of trauma and reconstructive surgery.

